# Isotropic and Anisotropic Monolayer Structures in RF Discharge Plasma

**DOI:** 10.3390/molecules28073259

**Published:** 2023-04-06

**Authors:** Anastasiya A. Alekseevskaya, Elena V. Vasilieva, Anatoly V. Filippov, Mikhail M. Vasiliev, Oleg F. Petrov

**Affiliations:** 1 Joint Institute for High Temperatures, Russian Academy of Sciences, Izhorskaya St. 13 Bld. 2, Moscow 125412, Russia; 2 Troitsk Institute for Innovation and Fusion Research, Pushkovykh St., vl. 12, Troitsk 108840, Russia

**Keywords:** colloidal plasma, two-dimensional structure, dusty plasma, RF discharge, anisotropy

## Abstract

We present the results of an experimental and analytical study of the structural and dynamic properties of a monolayer consisting of dust grains in an electrostatic trap in an RF discharge plasma. The possibility of forming a monolayer with an isotropic distribution for interparticle distance and kinetic energy of particles in the structure has been experimentally shown. Isotropy has crucial importance for the study of various processes in such systems, including the kinetics of phase transitions, the formation of directed flows, wave propagation, and others.

## 1. Introduction

Recently, there has been a surge of interest in active matter studies, including research of systems consisting of active Brownian particles in colloidal plasma. Active Brownian particles are particles capable of converting the energy of an external source into the energy of their own (not thermal) motion [1,2,3]. Colloidal plasma is an open dissipative system in which charged granules of micron and submicron sizes levitate in plasma and, due to strong electrostatic interactions, form ordered structures of various types depending on conditions: chains, vortices, self-oscillatory systems, crystalline-like structures, etc. Such systems can be studied at the kinetic level by observing and analyzing the motion of individual particles of the system. The fundamental property of such systems is their ability to self-organize [4], namely, the formation of collective motions (vortices [5], solitons [6], etc.), or evolution of structures, passing through transformations during non-equilibrium phase transitions [7,8,9]. To keep like-charged granules from scattering, a metal ring laying on the electrode is usually used, which creates an electrostatic trap (confinement). This confinement can significantly affect the properties of systems. For the first time, density inhomogeneity for systems of particles, whose interaction is described by a screened Coulomb potential was discussed in [10,11]. In [12], the influence of a parabolic trap on the radial distribution of the interparticle distance in the system was discussed. In a number of numerical and theoretical papers [12,13,14,15,16,17,18], the authors consider that radial inhomogeneity of a dust monolayer (namely, the interparticle distance at the periphery of the structure is greater than at its center) is a fundamental feature. Consequently, the term “dusty plasma crystal” is questioned as applied to ordered systems of dust particles in the plasma of gas discharges.

In this paper, we present the experimental results confirming the fact that the interparticle distance in a dusty monolayer can widely vary, depending on the parameters of the gas discharge. It can be heterogenic, both with a compact center and more rarefied periphery, as well as with a loose packing of particles in the center and a closely packed periphery. Moreover, it can be isotropic as well over the entire diameter of the structure under certain conditions. This fact is of crucial importance and should be taken into account when designing experimental studies of various processes in such systems, including the kinetics of phase transitions, the formation of directed flows, the development of an oscillatory process, etc. In this case, one should first set the discharge parameters to obtain a monolayer with isotropic properties, and then, without changing the discharge parameters, affect the properties of the structure by applying an external perturbation, for example, laser radiation in the case of kinetic heating of active Brownian macroparticles [19].

## 2. Results and Discussion

### 2.1. Experimental Results

**Figure 1:**
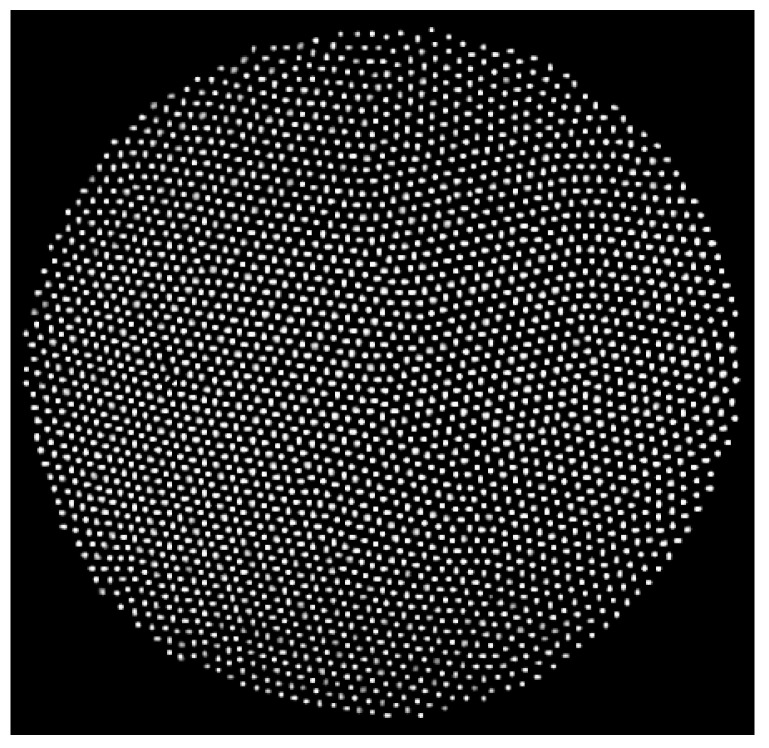
Video frame of isotropic dust monolayer structure in plasma of RF discharge at W = 6.9 W, P = 5 Pa.

Figure 1 shows a video frame of a monolayer structure of charged particles in RF discharge plasma. The evolution of the structure was experimentally observed while changing the discharge parameters. Based on the particle coordinates reconstructed from the video recording, we obtained the radial distribution of interparticle distances in the structure.

**Figure 2:**
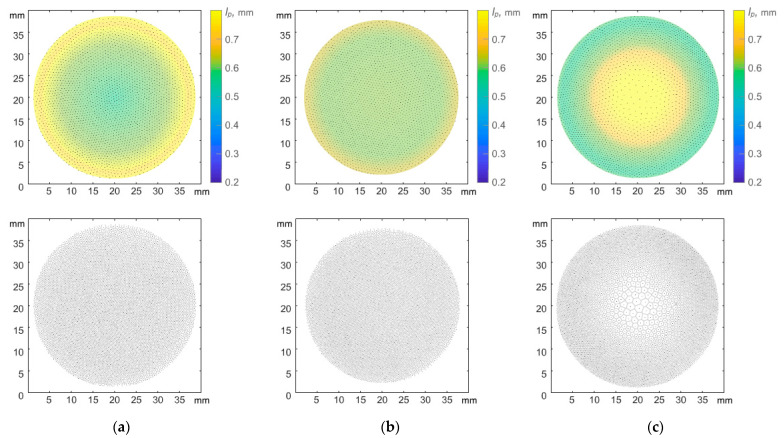
Quasi-two-dimensional dust structures in argon plasma of RF discharge at P = 5 Pa and various values of discharge power W: (**a**) 4.4 W, (**b**) 6.9 W, (**c**) 16.7 W. Color bar shows radial distribution of interparticle distances (upper row), Voronoi diagrams (lower row) represent different degrees of structure inhomogeneity.

It was experimentally observed that a change of discharge power led to a change of interparticle distance under a constant value of plasma-forming gas pressure P = 5 Pa (see Figure 2).

**Figure 3:**
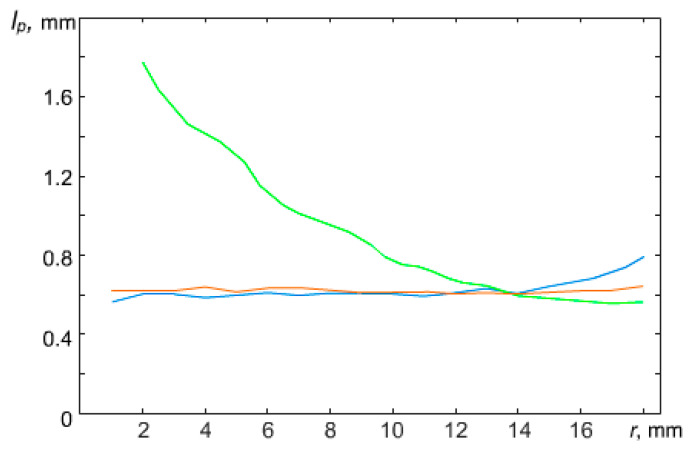
A dependence of the radial distribution of the average interparticle distance in the quasi-two-dimensional structure in an argon plasma of RF discharge at P = 5 Pa and various values of discharge power W: blue curve is for W = 4.4 W; red is for 6.9 W; green is for 16.7 W.

At a power of W = 4.4 W, the interparticle distance in the central part of the structure appeared to be about 1.5 times less than the interparticle distance at the periphery of the structure. However, with increasing power, the dust monolayer changed and at W = 6.9 W, the interparticle distances in the structure leveled off. A further increase in the discharge power to W = 16.7 W led to the formation of a sparse area in the central part of the structure, where the interparticle distances were significantly greater than at the periphery of the structure (Figure 3). A further increase in power led to the formation of a void in the central part of the structure. The appearance of voids in dusty plasma structures was previously observed in [20,21,22,23].

**Figure 4:**
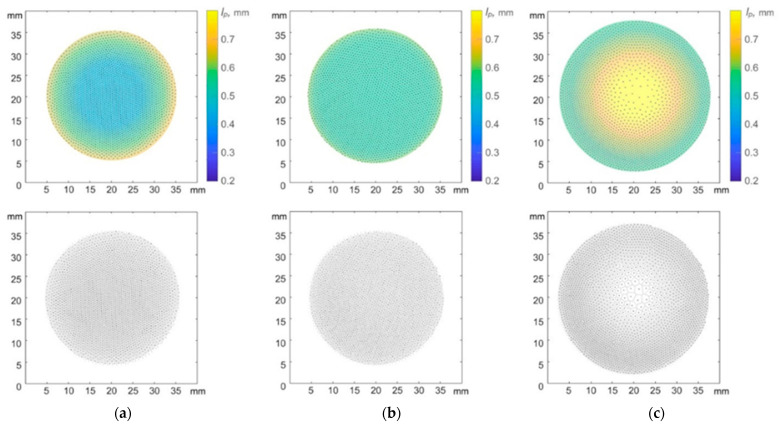
Quasi-two-dimensional dust structures in argon plasma of RF discharge at W = 13.1 W and various values of argon pressure P: (**a**) 3 Pa, (**b**) 4 Pa, (**c**) 5 Pa. Color bar shows radial distribution of interparticle distances (upper row), Voronoi diagrams (lower row) represent different degrees of structure inhomogeneity.

**Figure 5:**
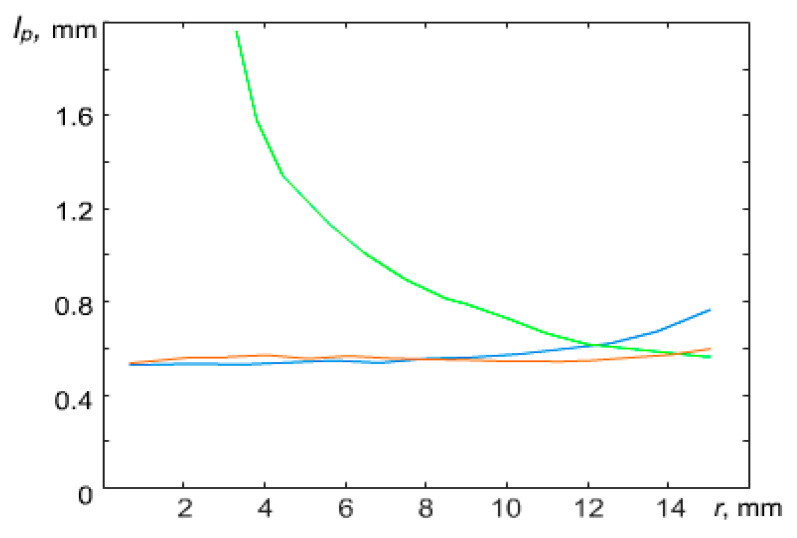
A dependence of the radial distribution of the average interparticle distance in the quasi-two-dimensional structure in an argon plasma of RF discharge at W = 13.1 W and various values of argon pressure P: (a) blue curve is for 3 Pa, (b) red is for 4 Pa, (c) green is for 5 Pa.

A similar trend was observed with a fixed discharge power W = 13.1 W while varying argon pressure in the discharge chamber (Figure 4). At P = 3 Pa, the interparticle distance in the central part of the structure was about 2 times less than the interparticle distance at the periphery of the structure. With an increase in pressure to P = 4 Pa, the interparticle distances in the structure were equalized. A further increase in pressure to P = 5 Pa led to the formation of a sparse area in the central part of the structure (Figure 5).

In work [12], the effect of a parabolic trap on the confined dusty plasma structure of particles interacting by a screening Coulomb potential was theoretically investigated. The authors came to a conclusion, based on the simplified model of dusty plasma, that the structures should be always heterogeneous. However, one can observe various structure configurations, including homogeneous, in the laboratory experiment (see Figure 2 and Figure 4). In the next section, we present theoretical models which can explain the obtained experimental results.

### 2.2. Basic Equations of the Hydrodynamic Model for Dusty Plasma in a Capacitive RF Discharge

The electron component in the dust plasma of an RF discharge can be described by the equations of the balance of the particles’ number and electron energy in terms of a hydrodynamic approximation with the values, such as electron concentration, average energy, and drift velocity, averaged over the period of the RF field (the derivation of these equations is presented, for example, in [24,25,26]):

(1) $$\frac{\partial n_{e}}{\partial t}+\mathrm{div}j_{e}=k_{ion}n_{e}N-J_{d}n_{d}$$

(2) ∂(ne〈ε〉)∂t+divhe=eEp⋅grad(neDe)+eμeneEp2+12eμe,ReneE02−−neNWel−kexneNEex−kionneNEion−13〈ε〉Jdnd,

where *n_e_* is the electron number density, **j***_e_* is the electron flux density in the drift-diffusion approximation defined by

$$j_{e}=-\mathrm{grad}\left(n_{e}D_{e}\right)-\mu_{e}n_{e}E_{p},$$

*D_e_*, *μ_e_* are the electron diffusion coefficient and the mobility, respectively, **E***_p_* is the strength of the polarization constant field of the electrode sheaths, *k_ion_* is the total gas ionization coefficient, *J_d_* is the total sink of electrons and ions on a dust particle, *n_d_* is the number density of dust particles, the *N* is the number density of atoms, 〈*ε*〉 is the mean electron energy, **h***_e_* is the electron energy flux density defined as

$$h_{e}=-\mathrm{grad}\left(n_{e}D_{\varepsilon }\right)-\mu_{\varepsilon }n_{e}E_{p}$$

*μ_e,_*_Re_ is the real part of mobility, in phase with the RF field, *μ_ε_* is the energy mobility, *D_ε_* is the energy diffusion coefficient, *W_el_* is the total net energy loss rate due to elastic electron-neutral collisions, *E_ion_* is the ionization potential, *k_ex_* is the atom excitation coefficient, *E_ex_* is the atom excitation potential.

We added to electron number balance Equation (1) a term that takes into account the absorption of electrons by dust particles, where an electron flux *J_e_* and an equal ion flux *J_i_* (since the characteristic charging times are much less than the characteristic times of the particle motion and the formation of dusty plasma structures) can be determined from orbital-motion-limited (OML) theory [27]:

(3) $$\begin{array}{l} J_{e}=\pi a^{2}n_{e}{\left(\frac{16\langle \varepsilon \rangle }{3\pi m_{e}}\right)}^{1/2}\exp\left(\frac{3}{2}\frac{e\phi_{d}}{\langle \varepsilon \rangle }\right),\\ J_{i}=\pi a^{2}n_{i}{\left(\frac{8T_{i}}{\pi m_{i}}\right)}^{1/2}\left(1-\frac{ez_{i}\phi_{d}}{T_{i}}\right), \end{array}$$

where *J_e_*, *J_i_*—the number of plasma electrons and ions absorbed by a dust particle of radius *a* per unit time, *ϕ_d_*—the potential of the particle surface, which can be found from the equality of the fluxes of electrons and ions:

(4) $$J_{e}\left(\phi_{d}\right)=J_{i}\left(\phi_{d}\right)\equiv J_{d}.$$

Other processes of loss (for example, recombination) and creation of electrons (for example, stepwise ionization with the participation of metastable excited atoms) can be neglected under conditions of low pressures and a low degree of ionization.

**Figure 6:**
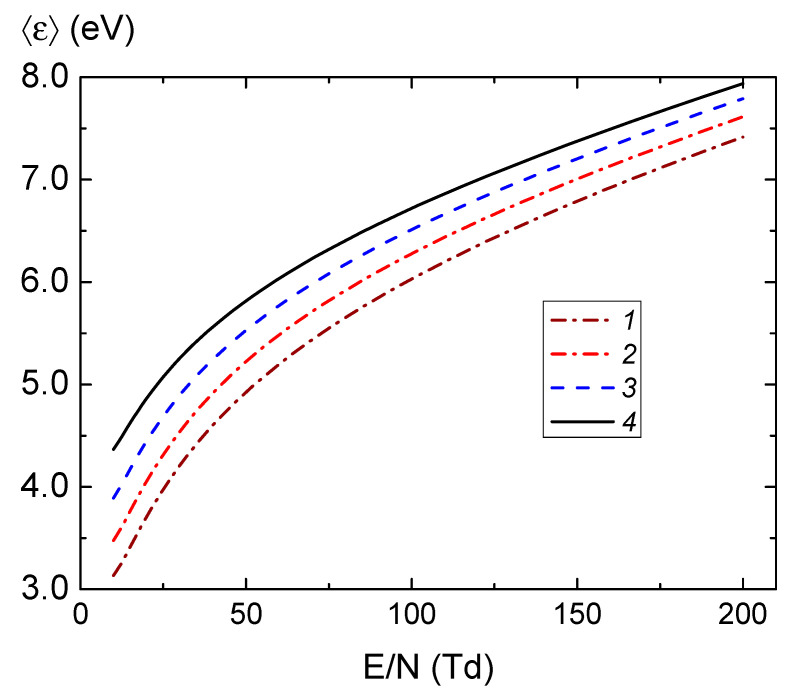
The dependence of the average electron energy on the specific intensity of the RF electric field with a frequency of 13.56 MHz in argon at pressure 1—3 Pa, 2—3.6 Pa, 3—4.5 Pa, 4—6 Pa.

**Figure 7:**
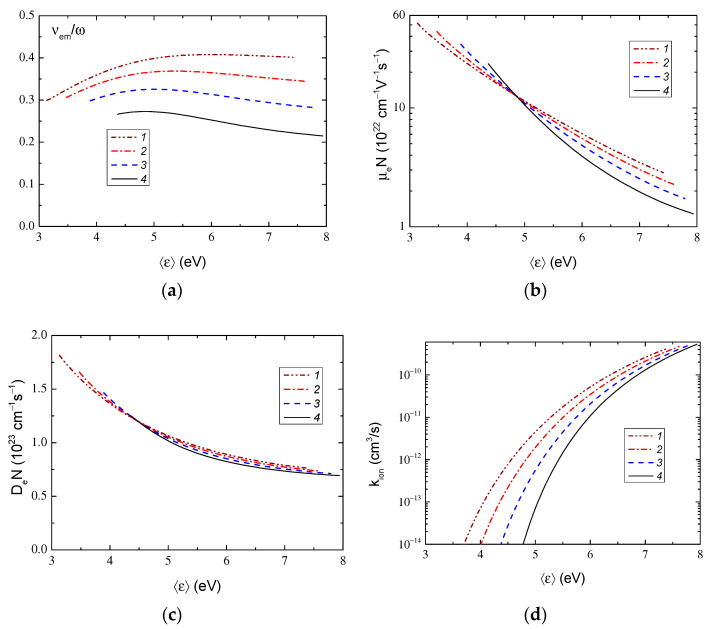
Dependences of the ratio of the averaged electron transport frequency to the circular frequency of the RF field (**a**), the mobility (**b**) and the electron diffusion coefficient (**c**), the ionization rate constant of atoms (**d**) on the average electron energy in the RF electric field with a frequency of 13.56 MHz in argon at pressure 1—3 Pa, 2—3.6 Pa, 3—4.5 Pa, 4—6 Pa.

The electron transfer coefficients and constants for ionization and excitation rates were calculated using the BOLSIG+ program [28,29] with cross sections from the LXCat set [30]. Figure 6 and Figure 7 show the calculated dependences of the average electron energy on the specific field and the electronic coefficients as functions of the average electron energy, respectively. Note that in Figure 6 $E=E_{0}/\sqrt{2}$, where *E*_0_ is the RF field amplitude. We also note that at the pressures indicated in the captions to the figures, the values of the specific circular frequency of the RF field *ω*/*N* form a uniform grid.

Figure 6 shows that the average electron energy increases with pressure growth and the ratio of the transport frequency *ν_m_* to the circular frequency of the RF field *ω* decreases (see Figure 7a). Note that the electron energy distribution function (EEDF) in a radio-frequency field when the condition *ω* ≫ *ν_u_* is met (*ν_u_* is the characteristic frequency of electron energy relaxation,$\nu_{u}\approx 2m_{e}\nu_{m}/M$, *m_e_* is the mass of the electron, *M* is the mass of the atoms of the working gas) is determined by the effective value of the field [26]:

(5) $$E_{eff}=\frac{\nu_{m}}{\sqrt{\omega^{2}+\nu_{m}^{2}}}\frac{E_{0}}{\sqrt{2}}.$$

Since the transport frequency depends on the energy of electrons, the effective field also depends on the energy of the electrons. The behavior of the average electron energy and the ratio *ν_m_*/*ω* noted above is a consequence of the complex behavior of the cross section of the collision of electrons with argon atoms, which passes through the deep Ramsauer minimum [30]. It can be seen in Figure 7 that the diffusion coefficient *D_e_* is a weak function of argon pressure (the values *μ_ε_*, *D_ε_*, and *W_el_* also had a similar behavior), and the electron mobility *μ_e_* and the ionization rate constant *k_ion_* noticeably change with pressure changes (the dependences *μ_e_*_,Re_, *k_exc_*, and *P_e_* were similar).

The polarization field of the near-electrode layers (so-called ambipolar field) is determined by the Poisson equation:

(6) $$\mathrm{div}E_{p}=4\pi e\left(z_{i}n_{i}-n_{e}+z_{d}n_{d}\right),$$

where *e* is the proton charge, *z_i_* is the charge number of ions (hereafter is assumed that *z_i_* = 1), *n_i_* is the ion number density, *z_d_* is the dust particle charge in elementary charges which is connected with the dust surface potential by relation

(7) $$z_{d}=\phi_{d}a\left(1+k_{D}a\right),$$

where *k_D_* is the inverse Debye radius.

The ion component is described by the equations of balance for ion number and flux density:

(8) $$\frac{\partial n_{i}}{\partial t}+\mathrm{div}j_{i}=k_{ion}n_{e}N-J_{i}n_{d},$$

where **j***_i_* is the ion flux density defined without allowance for the diffusion:

(9) $$j_{i}=\mu_{i}n_{i}E_{p},$$

*μ_i_* is the ion mobility.

**Figure 8:**
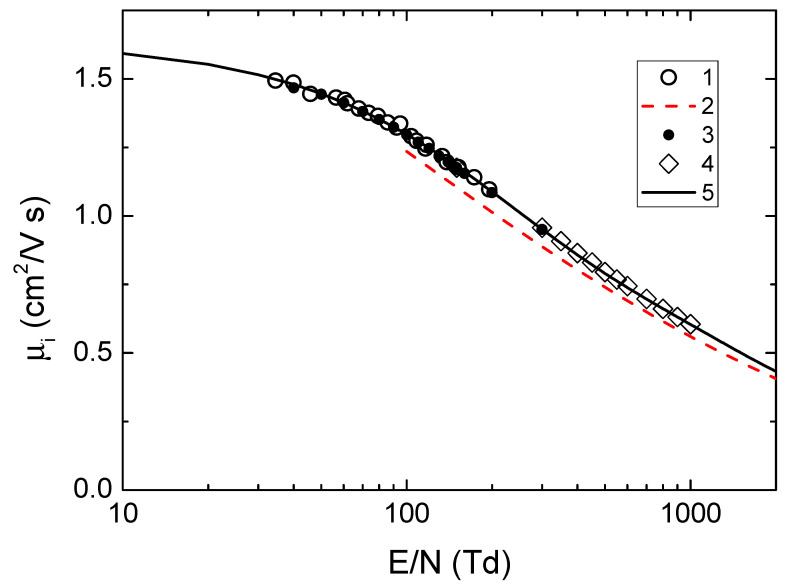
The mobility of Ar^+^ ions reduced to normal conditions in argon at a temperature of 300 K as a function of the reduced field E/N: 1—experimental data [29], 2—approximation from [30], 3—work [33], 4—[34], 5—approximation Equation (10).

The experimental mobilities of the argon ion (see Figure 8) via reduced field under normal conditions at a temperature of 300 K [30,31,32,33,34,35,36] are well approximated by the dependence:

(10) $$\mu_{i}=0.286+0.669\exp\left(-\frac{E/N}{179.5}\right)+0.679\exp\left(-\frac{E/N}{1305}\right).$$

Here *μ_i_* is valued in cm^2^/(V·s), and *E/N*—in Td.

Figure 8 shows only part of the literature data (more complete data are given in [32,33,34,35]). Note that in a number of papers (see, for example, [35]), to determine the ion drift velocity at high values of *E/N*, its relationship with the measured average ion energy *E_i_* in the form [36,37] was used:

(11) $$E_{i}=\frac{\pi m_{i}v_{i,dr}^{2}}{2},$$

where *m_i_* is the ion mass, v*_i,dr_* is the ion drift velocity. In our paper, we use the Wannier approximation [38,39],

(12) $$E_{i}=\frac{3}{2}T_{g}+\frac{\left(m_{i}+m_{g}\right)v_{i,dr}^{2}}{2},$$

where *m_g_* is the gas atom mass and *T_g_* is the gas temperature.

The dust component is described by the equations of the balance for the number of particles and the velocity of motion (see, for example, [40,41,42]):

(13) $$\begin{array}{l} \frac{\partial n_{d}}{\partial t}+\mathrm{div}\left(n_{d}v_{d}\right)=0;\\ \frac{\partial v_{d}}{\partial t}=g+\frac{1}{m_{d}}\left(ez_{d}E_{p}+F_{id}-F_{nf}\right) \end{array}$$

where **g** is a free fall acceleration, **F***_id_* is an ion drag or the force, induced by ion flux focusing, **F***_nf_* is the gas resistance force, which is determined at low pressures under the assumption of complete accommodation of the momentum of the gas atoms by the Epstein formula [43]:

(14) $$F_{nf}=-\frac{4}{3}\pi a^{2}\delta MNv_{g}v_{d},$$

v*_g_*—thermal velocity of gas atoms: $v_{g}=\sqrt{8T/\pi M},$, *δ* ≈ 1 is a coefficient depending on the nature of the reflection of gas atoms from the surface of a dust particle after a collision.

In [27] (see also [44,45] and references therein), the expression for the ion drag force is the following:

(15) $$F_{id,\varphi }=n_{i}v_{s}m_{i}v_{i}\pi \left(b_{c}^{2}+4b_{L}^{2}\Lambda \right),$$

where v*_Ti_*, v*_s_*, Λ, *b_L_*, *b_c_*, *R_De_* is thermal and total ion velocities, taking into account the drift motion, the Coulomb logarithm, the Landau radius, the ion capture radius, and the electron Debye radius, respectively, defined by the expressions:

(16) $$\begin{array}{c} v_{Ti}^{2}=\frac{8T_{i}}{\pi m_{i}},\;v_{s}^{2}=v_{i}^{2}+v_{Ti}^{2},\;\Lambda =\frac{1}{2}\ln(\frac{R_{d}^{2}+b_{L}^{2}}{b_{c}^{2}+b_{L}^{2}}),\\ b_{L}=\frac{e^{2}\left|z_{d}\right|}{m_{i}v_{s}^{2}},\;b_{c}=a\left(1-\frac{2e\phi_{d}}{m_{i}v_{s}^{2}}\right),\;R_{D}^{2}=\frac{\langle \varepsilon \rangle }{6\pi e^{2}n_{e}}. \end{array}$$

In this work, to study the dynamics of dust particles, we conducted simulations in a one-dimensional approximation along the *z* axis, directed upward towards gravity. As a first approximation, we assumed that the electron transfer coefficients and the gas ionization rate constant were not changing. This was justified, since in the regime under consideration, the field in the positive column was weak, and the electron density in the near-cathode layers was small.

In the steady state, the gas velocity can be set equal to zero, since the gas heating rate in the considered low-current RF discharge glowing mode was small. Additionally, the characteristic time for the establishment of the velocity of dust particles significantly exceeded the characteristic time for the establishment of their concentration; therefore, the inertia of dust particles can be neglected and their velocity can be determined from the stationary equation of motion of dust particles.

Let us show that the ion drag force can be comparable in magnitude to the force of gravity. In this case, taking into account the ion drag force is of fundamental importance, since it is responsible for changing the distribution of dust particles along the radius of the RF discharge.

### 2.3. Analytical Solution of RF Discharge

There are many works in the scientific literature devoted to the analytical approximate solution of an RF discharge (see, for example, [46,47,48,49,50]), but all of them are not free from shortcomings and have a limited area of applicability, which either lies in the region of rare collisions, or in the region of frequent collisions. In our experiments, we observe the case when the parameter *α_c_*, which determines the collisional or collisionless nature of ion transport in the near-electrode layers, defined by the relation [48]

(17) $$\alpha_{c}=\frac{\pi R_{De}}{l_{i}},$$

is valued as the order of unity (see Tables). Here, *R_De_* is the electron Debye radius, *l_i_* is the mean free path of ions. Therefore, we propose the following method for determining the parameters of an RF discharge under our conditions.

Let us consider the stationary solution of Equations (1), (6), and (8) for a symmetric RF discharge near the center of the discharge gap, where the polarization field is still sufficiently small and, therefore, dust particles cannot levitate. Let us assume that the transfer coefficients and the frequency of gas ionization in this region are constant. Equations (1), (6), and (8) for a one-dimensional problem take the form:

(18) $$\begin{array}{l} -D_{e}\frac{\partial^{2}n_{e}}{\partial z^{2}}-\mu_{e}\frac{\partial \left(n_{e}E_{p}\right)}{\partial z}=\nu_{ion}n_{e},\\ \mu_{i}\frac{\partial \left(n_{i}E_{p}\right)}{\partial z}=\nu_{ion}n_{e},\\ \frac{\partial E_{p}}{\partial z}=4\pi e\left(z_{i}n_{i}-n_{e}\right). \end{array}$$

From Equation (18), after simple algebra we find

(19) $$\frac{\partial E_{p}^{2}}{\partial z^{2}}=8\pi e\left[\left(\frac{1}{\mu_{e}}+\frac{1}{\mu_{i}}\right)\nu_{ion}n_{e}+\frac{D_{e}}{\mu_{e}}\frac{\partial^{2}n_{e}}{\partial z^{2}}\right].$$

We choose the origin of the *z* axis at the center of the discharge gap. Now, we seek a solution for the electron density in the form (see, for example, [51]):

(20) $$n_{e}=n_{e0}\left(z\right)\cos\left(\alpha z\right),$$

where *α* is an unknown constant. We are interested in a solution near the center of the discharge gap, so we assume that in this region *n_e_*_0_ is a weak function of the z coordinate and neglect its derivatives. Now from (19), using the discharge symmetry condition and the zero value of the field in the center, we find:

(21) $$E_{p}^{2}=8\pi e\left[\frac{1}{\alpha^{2}}\left(\frac{1}{\mu_{e}}+\frac{1}{\mu_{i}}\right)\nu_{ion}-\frac{D_{e}}{\mu_{e}}\right]n_{e0}\left[1-\cos\left(\alpha z\right)\right].$$

From Equation (14), using (16), we can find:

(22) $$\begin{array}{l} n_{e}E_{p}=\left(\frac{D_{e}\alpha }{\mu_{e}}-\frac{\nu_{ion}}{\alpha \mu_{e}}\right)n_{e0}\sin\left(\alpha z\right),\\ n_{i}E_{p}=\frac{\nu_{ion}}{\alpha \mu_{i}}n_{e0}\sin\left(\alpha z\right). \end{array}$$

From here, for the ratio of the concentrations of electrons and ions near the center of the discharge, we obtain:

(23) $$\frac{n_{e}}{n_{i}}=\frac{\alpha \mu_{i}}{\nu_{ion}}\left(\frac{D_{e}\alpha }{\mu_{e}}-\frac{\nu_{ion}}{\alpha \mu_{e}}\right)=\frac{\mu_{i}}{\mu_{e}}\left(\frac{D_{e}\alpha^{2}}{\nu_{ion}}-1\right).$$

Since there must be *n_e_ ≈ n_i_*, near the center of the RF discharge for the formation of a quasi-neutral plasma, then from (23) we find that for this equality the following expression must be satisfied:

(24) $$\frac{D_{e}\alpha^{2}}{\nu_{ion}}=1+\frac{\mu_{e}}{\mu_{i}}.$$

In general, the value of *α* is not defined here, but it can be expected that it will be of the order of 2*π*/*L,* where *L* is the inter-electrode distance. In this case, the commonly used zero boundary conditions for the electron concentration at the electrodes will be satisfied. Then the condition for the formation of a quasi-neutral plasma at the center of the RF discharge will have the form:

(25) $$\zeta \equiv \frac{\mu_{i}}{\mu_{e}}\left(\frac{4\pi^{2}D_{e}}{\nu_{ion}L^{2}}-1\right)=1.$$

Note that although we have four equations for determining *n_e_*, *n_i_*, and *E_p_* at the center of the discharge, but due to their approximate nature, they do not allow us to determine the value of the parameter *α*. Expression (25) allows one to determine the value of the reduced field *E*/*N* for which this condition is met. Further, the value of this field will be referred to as *E*_RF_.

**Figure 9:**
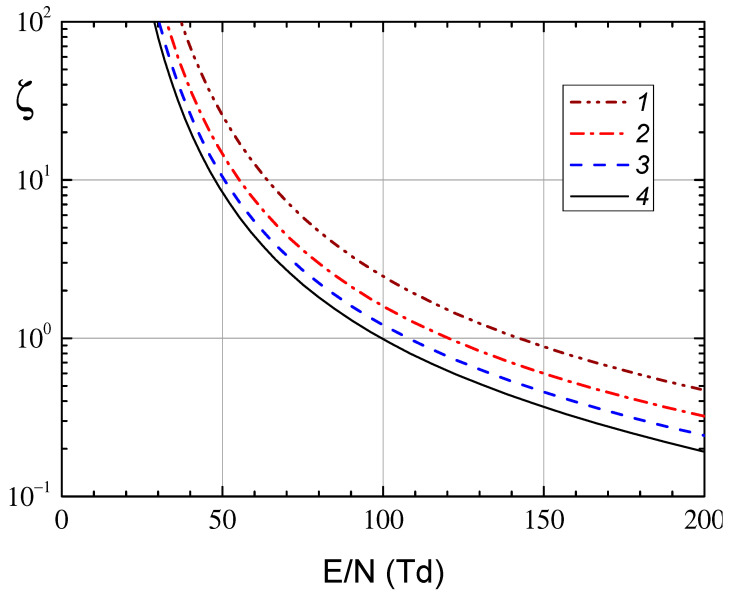
A dependence of parameter ζ on a reduced field E/N in RF discharge with a frequency of 13.56 MHz and inter-electrode distance L = 5 cm at various argon pressures: curve 1—3 Pa, curve 2—4 Pa, curve 3—5 Pa, curve 4—6 Pa.

Figure 9 shows the values of the parameter *ζ* as a function of the reduced field *E/N* at different values of the argon pressure. It can be seen from Figure 9 that the field required to ignite the RF discharge decreases with increasing pressure, which is associated with a decrease in the frequency of diffusive electron escape.

The power of the energy deposition into the RF discharge is the sum of the electronic and ionic parts, which can be written as [48]:

(26) $$W_{d}=W_{e}+W_{i}=\frac{1}{2}\left(V_{p}I+R_{sh}I^{2}\right),$$

where *R_sh_* is an ohmic resistance of the near-electrode layers, for which we use the relation *R_sh_S_HF_* ≈ 10^4^ Ω cm^2^ [48], *S_HF_* is an electrode area, *V_p_* is the ohmic part of the RF voltage across the RF discharge plasma, which also takes into account the so-called stochastic heating of electrons due to their reflection in the near-electrode layers. At this stage, we neglect stochastic heating, since there are debates in the literature about the mechanism of this heating, the analytical model of which is developed in [46,47,48] (see, for example, [51,52,53,54,55,56]).

Since the thickness of the near-electrode layers is several electron screening radii, which is negligibly small compared to the length of the discharge gap *L*, we take for estimates

(27) $$V_{p}=E_{0}L{\left(1+\frac{\omega^{2}}{\nu_{m}^{2}}\right)}^{-1/2},$$

Here *E*_0_ is amplitude of RF field. Then, knowing a value of the field *E*, found from the condition (25), and the experimental input power of the RF field *W_d_*, one can determine the RF discharge current and obtain the following expression for the ratio of ion and electron heating:

(28) $$\eta \equiv \frac{W_{i}}{W_{e}}=\frac{1}{2}\left(\sqrt{1+\frac{4R_{sh}W_{d}}{E^{2}L^{2}}\frac{\left(\nu_{m}^{2}+\omega^{2}\right)}{\nu_{m}^{2}}}-1\right).$$

As a result, we derive the following equation to determine the electron number density:

(29) $$n_{e}=\frac{W_{d}}{P_{e}\left(1+\eta \right)},$$

where *P_e_* is the energy per unit time absorbed by the electrons from the electric field: *P_e_*/*N* = (*μ_e_*_,Re_ *N*)(*E*/*N*)^2^. Table 1, Table 2, Table 3, Table 4 and Table 5 show the parameters of the plasma in the RF discharge and the dust component in the levitation region, where the condition is met:

(30) $$ez_{d}E_{p}=m_{d}g.$$

This field will be further denoted as *E*_lev_. The coefficients related to the electron component in Table 1, Table 2, Table 3 and Table 4 are determined by the value of the reduced *E/N* field found from (25), and the electron number density is determined from (29). It is assumed that the number density of ions is equal to the number density of electrons. The ion energy in the levitation region of dust particles *E_l_*_,ion_ is determined from the expression (12), while the drift velocity is determined using a field value equal to $\sqrt{E^{2}+E_{\mathrm{lev}}^{2}}$. This value of ion energy is used to determine the potential of dust particles *ϕ*_d_ from the transcendental Equation (4) and their charge *z_d_* from the ratio (7).

Table 3 and Table 4 also show the values of the charge *z_d_*_0_ and the floating potential of dust particles *ϕ*_0_, determined with the effective ion temperature: $T_{i}=\frac{2}{3}E_{i}$, where *E_i_* is the ion energy determined by Formula (12) taking into account only the drift motion in the RF field. It can be seen that this leads to a noticeable decrease in the charge of dust particles (in absolute magnitude). This is a consequence of the fact that the ion flow to a dust particle at high values of grain charge depends on the ion temperature as $T_{i}^{-1/2}$, since the last multiplier in Equation (3) becomes of order of unit. As a result, when taking into account the ion drift only in the RF field, the effective temperature turns out to be lower, and therefore, the ion flux increases. In order to maintain its balance with the electron flux, the potential of the dust particle must decrease in absolute magnitude. As can be seen from Table 5, the electric polarization field in the levitation region of the dust particles significantly exceeds the intensity of the RF field.

Table 5 shows the specific values of the power of energy deposition and stochastic heating, the ion drag force, the transport frequency, and the ratios of the ion and electron plasma frequencies to the circular frequency of the high-frequency field in the experiments described above. It can be seen that the ion drag force is only slightly lower than the gravity force and should be taken into account when determining the levitation height of dust particles. These assumptions will be considered in our future work, together with other RF discharge parameters determined from the numerical solution of the system of Equations (1), (2), (6), (8), and (13). In addition, one can see from Table 5 that the electron plasma frequency turns out to be much higher than the circular frequency of the RF field in our experiments, while the ion frequency is much lower. These conditions are considered in the analytical theory, developed in [48].

The power of stochastic heating of electrons in Table 5 was determined according to [51]:

(31) Wst=38nevTth,ee(u˜evth,e)2HGk(H),

where ${\tilde{u}}_{e}$ is the electron drift velocity amplitude, v*_th,e_* is the electron thermal velocity: $v_{th,e}=\sqrt{8T_{e}/\pi m_{e}}$, ${\tilde{s}}_{e}$ is the amplitude of electron oscillations in the bulk plasma, *H* is the parameter defined by the relation $H={\tilde{s}}_{e}^{2}/\pi R_{D}^{2}$, *G_k_*(*H*) is an integral that is well approximated by [52]

(32) $$G_{k}\left(H\right)\approx \frac{24}{H+55}.$$

The amplitudes of the drift velocity and oscillations in the RF field were determined from the ratios:

(33) u˜e=Eω2μe.Re2+μe.Im2,s˜e≈u˜eω.

**Figure 10:**
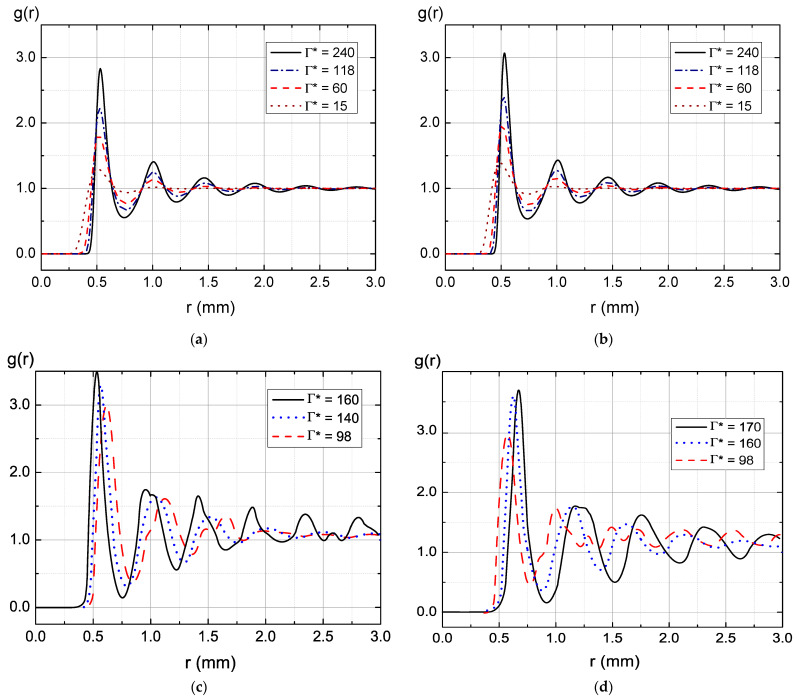
Pair correlation functions *g*(*r*), (**a**,**b**) determined by numeric solution of the Ornstein–Zernike equation in HNC approximation under various values of effective coupling parameters Γ* and *n_d_* = 8.0 × 10^3^ cm^−3^, and (**c**,**d**) obtained in experiments under the following parameters: (**a**) *η* = 5.53, *R_D_* = 1/*k_D_* = 293.3 μm, *λ* = 1.7, *z_d_* = −1687.7; (**b**) *η* = 0 (*W_i_* = 0), *R_D_* = 113.4 μm, *λ* = 4.4, *z_d_* = −3420.6; (**c**) Discharge power *W* = 13.1 W, pressure *P* = 3–5 Pa; (**d**) Discharge power *W* = 4.4–16.7 W, pressure *P* = 5 Pa.

With the values of the screening radius found, the pair correlation function (PCF) of dust particles was calculated for different charges or effective coupling parameters [57]:

(34) Γ*=ez2deadTd(1+kDad+12kD2ad2)e−kDad,

where *a_d_* is the mean interparticle distance of the dust particles: $a_{d}=n_{d}^{-1/3}$. Figure 10a shows the dependence of the PCF on the distance found by the numerical solution of the Ornstein–Zernike (OZ) iteration method [58,59] in the hypernetted chain (HNC) approximation. The figure caption indicates the charge value at Γ* = 240. The charge of the dust particles shown in Table 5 is an order of magnitude higher than this value. As can be seen from Figure 10, the height of the first peak increases with the growth of the charge, so a slight difference in the height of the experimental first peak may be due to the higher value of particle charge. Unfortunately, the iterative method of solving the OZ equation in the HNC approximation ceases to converge at higher values of the coupling parameter.

The determination of the fraction of the RF discharge power associated with the ionic component is approximate, so the pair correlation function is also calculated at zero ionic power *η* = 0 (see Figure 10b). In this case, the electron number density increases more than 6 times, while the Debye radius decreases and the value of the structural parameter *λ* = *k_D_a_d_* grows from 1.7 to 4.4. Since in numerous experiments on the study of dusty plasma, the value of the structural parameter does not exceed two, we can say that taking into account the ionic component of the power gives a more consistent result when compared with the experimental data.

The dependences of the PCF on the distance shown In Figure 10 are similar to those determined experimentally, which allows us to conclude that the analytical approach developed in this work is applicable for estimating the parameters of dusty plasma in an RF discharge. As can be seen from Table 5, according to analytical estimates, the force of ion drag in the experimental conditions turns out to be comparable to gravity; therefore, it must be taken into account when finding conditions for the levitation of dust particles.

## 3. Experimental Setup

**Figure 11:**
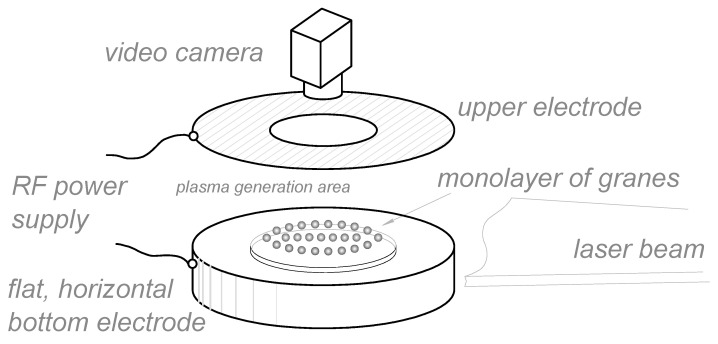
A scheme of an experimental setup.

For the experimental study of quasi-two-dimensional structures of charged macroparticles formed in the RF discharge plasma, an experimental setup described in [19] was used (see Figure 11). The main element of the setup is a vacuum chamber with optical windows in the horizontal plane and in the upper part of the chamber. The atmosphere is pumped out from the chamber by a consequently connected fore-vacuum and turbomolecular pumps to a residual pressure of 10^−4^ Pa. Then, the chamber is filled with plasma-forming gas—argon, up to a working pressure of 3–5 Pa. In the center of the chamber, there are two flat horizontal electrodes, the distance between which is 5 cm. A voltage of 350 V with a frequency of 13.56 MHz is applied to them, as a result of which low-pressure plasma ignites in the chamber.

After that, melamine–formaldehyde (MF) grains with a diameter of d = 10.6 μm and a density of ρ = 1.51 g/cm^3^ are injected into the discharge. To create an electrostatic trap, a ring 8 cm in diameter and 0.2 cm in height is installed on the lower electrode, so that it keeps the charged dust grains from scattering in the radial direction. Being illuminated by a flat horizontal beam of an argon laser, the grains can be easily seen with naked eye. We use the CCD—camera placed above the chamber to record top view of the structure in dynamics. The obtained video data is processed then using a specialized program code, as a result of which various characteristics of dust particles can be obtained, such as their coordinates and motion trajectories, pair correlation functions, the coupling parameter, etc.

Two series of experiments with a monolayer structure were carried out. In the first one, we fixed the working gas pressure at 5 Pa and varied the discharge power from 4.4 to 16.7 W. In the second series, we fixed the discharge power at the level of 13.1 W and varied the argon pressure from 3 to 5 Pa. Then, we processed the corresponding video recordings in order to obtain the above-mentioned parameters of the dusty plasma structure, and therefore, to calculate the radial density distribution of dust particles depending on pressure and discharge power.

## 4. Conclusions

Our experiments unambiguously show that in the plasma of an RF discharge, it is possible to observe structures of charged grains not only rarefied at the periphery and dense in the center, but also with an isotropic interparticle distance, as well as more rarefied in the central part. This fact is of crucial importance and should be taken into account when designing experimental studies of various processes in such systems, including the kinetics of phase transitions, the formation of directed flows, the development of an oscillatory process, etc.

It is shown that by changing the discharge power or the pressure of the plasma-forming gas, one can influence the radial distribution of the interparticle distance in the dust monolayer and the uniformity of the structure.

Previously, based on the hydrodynamic model of dusty plasma in a capacitive RF discharge without taking into account the ion drag force, it was assumed that being placed in a parabolic trap, particles form a structure with a rarefied periphery. However, in this work, it is shown that the ion drag force can be comparable in magnitude to the force of gravity. The ion drag force is believed to be taken into account in the model, since it is responsible for changing the distribution of dust particles along the radius of the RF discharge. It is known that this force leads to the formation of voids in RF discharges, and in our experiments, the formation of voids also took place at certain plasma parameters.

Verification of the hypothesis about the influence of the ion drag force on the inhomogeneity in the distribution of dust particles requires more accurate calculations, which is planned to be done by self-consistent numerical simulation of dusty plasma in an RF discharge within the framework of the hydrodynamic model developed in this work.

## Figures and Tables

**Table 1:** Parameters of the RF discharge in the first series of experiments at the fixed discharge power (W_in_ = 15 W, W_out_ = 4.7 W) and at different pressures.

*P* (Pa)	*E*/*N* (Td)	*n_e_* (cm^−3^)	*T_e_* (eV)	*R_Di_* (μm)	*R_De_* (μm)	*l_e_* (cm)	*l_i_* (cm)
3	142.46	3.39 × 10^8^	4.457	379.4	851.9	2.304	0.078
4	120.45	2.95 × 10^8^	4.472	354.1	915.0	1.663	0.061
5	107.89	2.61 × 10^8^	4.478	336.7	974.1	1.297	0.050
6	99.66	2.34 × 10^8^	4.476	324.1	1028.4	1.063	0.042

**Table 2:** Parameters of the RF discharge in the second series of experiments at the fixed pressure P = 5 Pa (*E/N* = 107.9 Td, *T_e_* = 4.478 eV, *l_e_* = 1.297 cm, *l_i_* = 0.05 cm).

W_in_ (W)	W_out_ (W)	w*_e_* (eV cm^−3^ s^−1^)	*n_e_* (cm^−3^)	*R_Di_* (μm)	*R_De_* (μm)	*η = W_i_*/*W_e_*
30	4.60	4.03 × 10^15^	4.21 × 10^8^	264.5	767.0	10.14
20	3.30	3.24 × 10^15^	3.37 × 10^8^	295.7	856.4	8.13
8	1.10	2.01 × 10^15^	2.10 × 10^8^	375.5	1085.5	5.06
5	0.60	1.57 × 10^15^	1.64 × 10^8^	425.4	1228.6	3.95

**Table 3:** Parameters of the RF discharge and dust particles (with radius *r*_0_ = 5.3 μm and density *ρ* = 1.5 g/cm^3^) in the levitation area during the first series of experiments at the fixed discharge power.

*P* (Pa)	*ϕ*_0_ (V), *T_i_* = *T*_gas_	*z_d_*_0_ (*e*) *T_i_* = *T*_gas_	*ϕ*_d_ (V)	*z_d_* (*e*)	*E*_lev_ (V/cm)	*E* (V/cm)	*E_l,_*_ion_ (eV)
3	−9.906	−3.94 × 10^4^	−15.53	−5.81 × 10^4^	9.87	1.068	1.326
4	−9.934	−3.94 × 10^4^	−15.14	−5.66 × 10^4^	10.12	1.205	1.005
5	−9.945	−3.93 × 10^4^	−14.80	−5.54 × 10^4^	10.35	1.349	0.803
6	−9.941	−3.91 × 10^4^	−14.49	−5.43 × 10^4^	10.56	1.495	0.667

**Table 4:** Parameters of the RF discharge and dust particles (with radius *r*_0_ = 5.3 μm and density *ρ* = 1.5 g/cm^3^) in the levitation area during the second series of experiments at the fixed pressure.

w*_e_* (eV/cm^3^ s)	*ϕ*_0_ (V) *T_i_* = *T*_gas_	*z_d_*_0_ (*e*) *T_i_* = *T*_gas_	*ϕ*_d_ (V)	*z_d_* (*e*)	*E*_lev_ (V/cm)	*E* (V/cm)	*E_l,_*_ion_ (eV)
4.03 × 10^15^	−9.945	−3.99 × 10^4^	−14.79	−5.56 × 10^4^	10.31	1.349	0.799
3.24 × 10^15^	−9.945	−3.96 × 10^4^	−14.80	−5.55 × 10^4^	10.33	1.349	0.801
2.01 × 10^15^	−9.945	−3.90 × 10^4^	−14.80	−5.53 × 10^4^	10.36	1.349	0.804
1.57 × 10^15^	−9.945	−3.87 × 10^4^	−14.80	−5.52 × 10^4^	10.38	1.349	0.805

**Table 5:** Ion drag force, power of stochastic heating of electrons, transport frequency (ν_m_), and ratio of ion and electron plasma frequencies to the circular frequency of the RF discharge *ω* = 2π × 13.56 MHz = 8.52 × 10^7^ s^−1^.

P (Pa)	W_in_ (W)	w*_e_* (eV cm^−3^ s^−1^)	W_st_ (eV cm^−3^ s^−1^)	F_id_ (dyn) *	ν_m_ (s^−1^)	${\left(\frac{\omega_{pi}}{\omega }\right)}^{2}$	${\left(\frac{\omega_{pe}}{\omega }\right)}^{2}$
3	15	2.79 × 10^15^	1.23 × 10^16^	8.36 × 10^−7^	3.46 × 10^7^	2.04 × 10^−3^	149
4	15	2.62 × 10^15^	7.77 × 10^15^	7.51 × 10^−7^	2.80 × 10^7^	1.78 × 10^−3^	129
5	15	2.50 × 10^15^	5.32 × 10^15^	6.60 × 10^−7^	2.34 × 10^7^	1.57 × 10^−3^	114
6	15	2.41 × 10^15^	3.86 × 10^15^	5.74 × 10^−7^	2.00 × 10^7^	1.41 × 10^−3^	103
5	30	4.03 × 10^15^	1.02 × 10^15^	8.57 × 10^−7^	2.34 × 10^7^	2.53 × 10^−3^	184
5	20	3.24 × 10^15^	7.61 × 10^15^	7.64 × 10^−7^	2.34 × 10^7^	2.03 × 10^−3^	148
5	8	2.01 × 10^15^	3.89 × 10^15^	5.80 × 10^−7^	2.34 × 10^7^	1.26 × 10^−3^	92.0
5	5	1.57 × 10^15^	2.68 × 10^15^	4.96 × 10^−7^	2.34 × 10^7^	9.86 × 10^−3^	71.9

* *m_d_g* = *ez_d_ E*_lev_ *=* 9.17 × 10^−7^ dyn.

## Data Availability

Data sharing is not applicable to this article.

## Sample Availability

Samples of the compounds are not available from the authors.
